# Arthroscopically assisted internal fixation for treatment of acute ankle fracture: A systematic review and meta-analysis of comparative studies

**DOI:** 10.1371/journal.pone.0289554

**Published:** 2023-08-04

**Authors:** Chen Zhuang, Wenxuan Guo, Wenhuan Chen, Yu Pan, Rujie Zhuang

**Affiliations:** 1 Alberta Institute, Wenzhou Medical University, Wenzhou, Zhejiang, China; 2 Department of Orthopaedics, The First Affiliated Hospital of Zhejiang Chinese Medical University (Zhejiang Provincial Hospital of Traditional Chinese Medicine), Hangzhou, Zhejiang, China; 3 Quzhou Hospital of Traditional Chinese Medicine, Quzhou, Zhejiang, China; 4 Quzhou TCM Hospital at the Junction of Four Provinces Affiliated to Zhejiang Chinese Medical University, Quzhou, Zhejiang, China; Assiut University Faculty of Medicine, EGYPT

## Abstract

**Background:**

Arthroscopically assisted reduction and internal fixation (ARIF) allows for the assessment of joint congruity following anatomic reduction, identification of occult intra-articular lesions, and treatment of traumatic intra-articular pathologies. The aim of this systematic review and meta-analysis was to provide evidence on whether ARIF is an alternative treatment protocol for ankle fractures.

**Methods:**

The PubMed, Embase, and Cochrane Library databases were searched independently by two investigators from the inception dates to October 9, 2022, for comparative studies. The risk-of-bias tool of the Cochrane Collaboration for Randomized Controlled Trials and the methodological index for non-randomized studies (MINORS) were used for assessing the methodological quality. Outcomes were evaluated in terms of the Olerud–Molander Ankle Score (OMAS), American Orthopaedic Foot and Ankle Society (AOFAS) Ankle–Hindfoot Scale, post-operative complications, arthroscopic findings, Visual Analogue Scale (VAS) score, and operation time. Cochrane Review Manager Software 5.4 was used to perform the statistical analysis.

**Results:**

A total of 10 trials involving 755 patients were included in this meta-analysis. The results revealed that ARIF for ankle fractures was superior regarding functional outcomes and VAS scores when compared with open reduction and internal fixation (ORIF). No significant difference was noted in the post-operative complication rate and the operation time between the ARIF and ORIF groups. A high incidence of chondral or osteochondral lesions (OCLs), ligamentous injuries, and loose bodies with ankle fractures was found by ankle arthroscopy.

**Conclusions:**

ARIF for ankle fractures might be beneficial to offer superior functional outcomes and VAS score than ORIF. Orthopedic surgeons should take a high incidence of OCLs and ligamentous injuries into consideration for the treatment of acute ankle fractures. We believe that with the increase in surgical experience, the occurrence of post-operative complications and the extension of operation time will no longer be a potential concern for surgeons.

## Introduction

Ankle fracture is one of the most common injuries, often accompanied by cartilage lesions and ligament injuries [1]. The standard treatment protocol for unstable ankle fractures is open reduction and internal fixation (ORIF) [2, 3]. Despite receiving ORIF and achieving perfect anatomical reduction, some patients still complain residual persistent pains and unsatisfactory functional outcomes [4–7]. Approximately 1% of patients with ankle fractures develop end-stage ankle osteoarthritis after ORIF and undergo total ankle replacement or ankle arthrodesis [8].

The residual pain and progression of osteoarthritis may be ascribed to concomitant intra-articular injuries occurring at the time of the initial fracture [9, 10]. The incidence of intra-articular injuries associated with rotational ankle fractures is as high as 63%–79% [11, 12]. A systematic review reported the incidence of chondral or osteochondral lesions (OCLs) identified by ankle arthroscopy after in rotational ankle fracture to be 54.5% [13]. These OCLs are believed to contribute to residual pain, dysfunction, locking, and early arthritis of ankle fractures [6]. Therefore, several groups of authors have emphasized the value of ankle arthroscopy in the treatment of acute fractures [7].

Arthroscopically assisted reduction and internal fixation (ARIF) allows for the assessment of joint congruity following anatomic reduction, identification of occult intra-articular lesions, and treatment of traumatic intra-articular pathologies [7, 14]. Despite the above-mentioned benefits, there is still a paucity of high-quality evidence to compare the clinical outcomes of ARIF with conventional ORIF.

Due to an increase in related studies that have been published in recent years, a systematic review and meta-analysis of comparative studies is imperative to provide evidence on whether ARIF is an alternative treatment protocol for ankle fractures. In this study, a systematic review and meta-analysis of comparative studies was performed to report the functional outcomes, arthroscopic findings, and the complication rate of ARIF for acute ankle fracture.

## Methods

This research was registered with the international prospective register of systematic reviews (PROSPERO, registration number: CRD42022330252). This systematic review and meta-analysis was performed in accordance with the Preferred Reporting Items for Systematic Reviews and Meta-analyses (PRISMA) guidelines [15]. A PRISMA checklist is provided in the S1 Checklist.

### Search strategy

PubMed, Embase, and Cochrane Library databases were searched independently by two investigators from the inception dates to October 9, 2022. We used the keywords “ankle,” “malleolus,” “fracture,” “arthroscopy,” “arthroscope,” and “endoscope.” In addition, we screened the reference lists of the included studies for additional relevant studies.

### Selection criteria

Studies were included based on the following criteria: (1) patients aged over 16 years with acute ankle fractures; and (2) clinical trials comparing ARIF versus ORIF. The exclusion criteria were as follows: (1) trials without available data; (2) studies not written in English; (3) arthroscopy in sequalae for ankle fractures; (4) posterior arthroscopy for ankle fractures; and (5) conference abstracts, experimental studies, and animal studies. The primary outcome was the functional outcome at the final follow-up time. Secondary outcomes were the rate of complications, arthroscopic findings, the Visual Analogue Scale (VAS) score, and operation time.

### Study selection and data extraction

Two independent researchers (C.Z. and W.-X.G.) screened the study titles and abstracts according to the inclusion criteria. The full text of the studies potentially meeting the eligibility criteria were retrieved for a detailed perusal to make a final decision regarding inclusion. The following data were extracted: lead author; publication year; country of origin; study design; sample size; age; follow-up duration; fracture type; functional outcome tools and other outcomes. In case of disputes, a third reviewer (W.-H.C.) was involved in the discussion to arrive at a consensus.

### Quality assessment

Two independent investigators (C.Z. and W.-X.G.) evaluated the quality of the included studies. The risk-of-bias tool of the Cochrane Collaboration for Randomized Controlled Trials (RCTs) was used by two independent reviewers to assess the methodological quality [16]. The seven items used to evaluate bias in each trial included the randomization sequence generation, allocation concealment, blinding of participants and personnel, blinding of outcome assessments, incomplete outcome data, selective reporting, and other biases, such as the baseline characteristics between different groups. The methodological quality of the non-randomized studies was assessed using the methodological index for non-randomized studies (MINORS) [17]. The MINORS tool consists of eight items for noncomparative and 12 items for comparative nonrandomized studies.

### Data analysis

All data management and meta-analyses were conducted using RevMan 5.4 software (Cochrane Collaboration, London, UK). The mean difference (MD) or standardized mean difference (SMD) was used as the effect analysis statistic for continuous variables. The risk ratio (RR) was used as the effect analysis statistic for categorical variables. The 95% confidence interval (CI) was calculated for each statistic. Statistical heterogeneity among the summary data was evaluated using the I^2^ statistic. For I^2^ ≤ 50%, the heterogeneity was considered not significantly different, and a fixed-effects model was used for the meta-analysis. In case of statistical heterogeneity among studies, the source of heterogeneity was further analyzed. After excluding the obvious source of clinical heterogeneity, a random-effects model was used to pool the data. When obvious clinical heterogeneity existed, the researchers performed subgroup or sensitivity analyses or only descriptive analyses. Study-specific and pooled estimates are graphically depicted by forest plots. The publication bias among various studies was assessed using the visual examination of funnel plot and Egger’s test if 10 or more studies were available [18]. A p-value < 0.05 was considered statistically significant.

## Results

**Fig 1:**
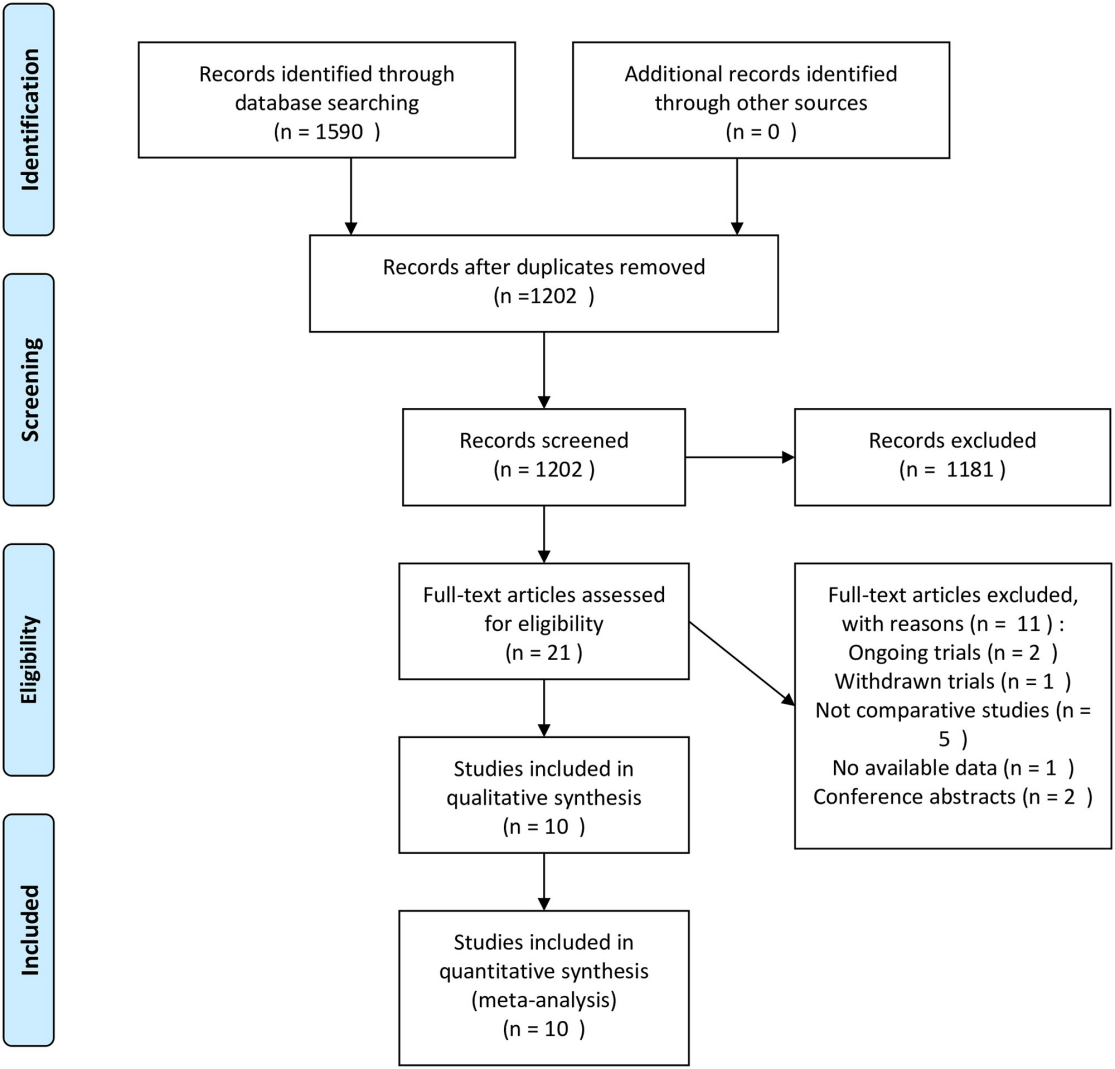
Flow diagram of study search and selection process.

Based on the searches of published articles, 1,590 potentially eligible records were identified. Of these, 1202 studies were screened by title and abstract after duplicate removal. With careful screening of the 21 full-text studies mentioned above was completed, 10 studies met the inclusion criteria and were retained [14, 19–27]. The remaining studies were excluded for various reasons. A flow diagram illustrating study searching and selection process is demonstrated in Fig 1.

### Baseline characteristics

Of the 10 included studies, three studies were RCTs [23, 25, 26] and seven trials were non-randomized comparative studies [14, 19–22, 24, 27]. A total of 755 patients with a mean age of 43.3 years and a mean follow-up period of 39.5 months were included in this systematic review. In total, 345 patients received ARIF at the time of fracture reduction, while 410 patients were received conventional ORIF. The baseline characteristics of the included studies are presented in Table 1.

**Table 1:** The baseline characteristics of the included studies.

Included Studies	Country	Study design	Sample size	Age (mean ± SD, years)	Follow-up (mean ± SD, months)	Fracture type, n	Functional outcome tools	Other outcomes
Angthong 2016	Thailand	retrospective cohort study	ARIF: 16	47.8±16.3	9.8(range, 4–22)	Supination-type, 33; Pronation-type, 12	NR	Complications Arthroscopic findings
			ORIF: 29	45.0±16.8				
Baumbach 2021	Germany	retrospective comparative study	ARIF:25	46/28(Median/IQR)	52.8/9.6(Median/IQR)	Weber A, 2; Weber B, 39; Weber C, 9	OMAS FAAM	Complications
			ORIF:25	53/22(Median/IQR)	48/40.8(Median/IQR)			
Chiang 2019	China	retrospective comparative study	ARIF: 65	45.4±19.47	40.02±13.40	Weber B, 105	AOFAS	Complications VAS Operation time
			ORIF: 40	49.5±19.3	38.44±14.47			
Fuchs 2016	USA	retrospective cohort study	ARIF: 24	38.3	67	Weber B, 33; Weber C, 18	PROMIS OMAS	Complications Arthroscopic findings VAS Operation time
			ORIF: 27	40.3				
Ge 2017	China	RCT	ARIF: 34	65.8±4.6	Not reported	Not reported	McGuire score	VAS Operation time
			ORIF: 34	66.5±5.2				
Liu 2020	China	retrospective comparative study	ARIF: 39	36.9±11.3	60.1±13.5	Isolated medial malleolar fracture, 85	OMAS	Complications Arthroscopic findings VAS Operation time
			ORIF: 46	38.1±10.4	61.5±11.2			
Smith 2020	USA	retrospective comparative study	ARIF: 71	39.9	32.4	Weber B, 158; Weber C, 55	PROMIS	Complications Arthroscopic findings Operation time
			ORIF: 142	40				
Takao 2004	Japan	RCT	ARIF: 41	36 (range, 20–64)	40 (range, 28–53)	Weber B, 72	AOFAS	Complications Arthroscopic findings
			ORIF: 31	38 (range, 20–58)	41 (range, 31–53)			
Thordarson 2001	USA	RCT	ARIF: 9	29	21 (range, 6–39)	SER, 16; PER, 3	SF-36 Foot and Ankle MODEMS	Complications
			ORIF: 10					
Turhan 2013	Turkey	retrospective comparative study	ARIF: 21	34±6.6	26(range, 18–52)	Isolated medial malleolar fracture, 47	OMAS	Complications Arthroscopic findings Operation time
			ORIF: 26	42±8.9				

NR, Not Reported; OMAS, Olerud and Molander Ankle Scores; AOFAS, American Orthopaedic Foot & Ankle Society; PROMIS, Patient Reported Outcome Measurement Information System; VAS, The visual analogue scale; SF-36, The MOS 36-ltem Short-Form Health Survey; MODEMS, Musculoskeletal Outcomes Data Evaluation and Management Systems

### Risk-of-bias assessments

#### Randomized controlled trials

**Fig 2:**
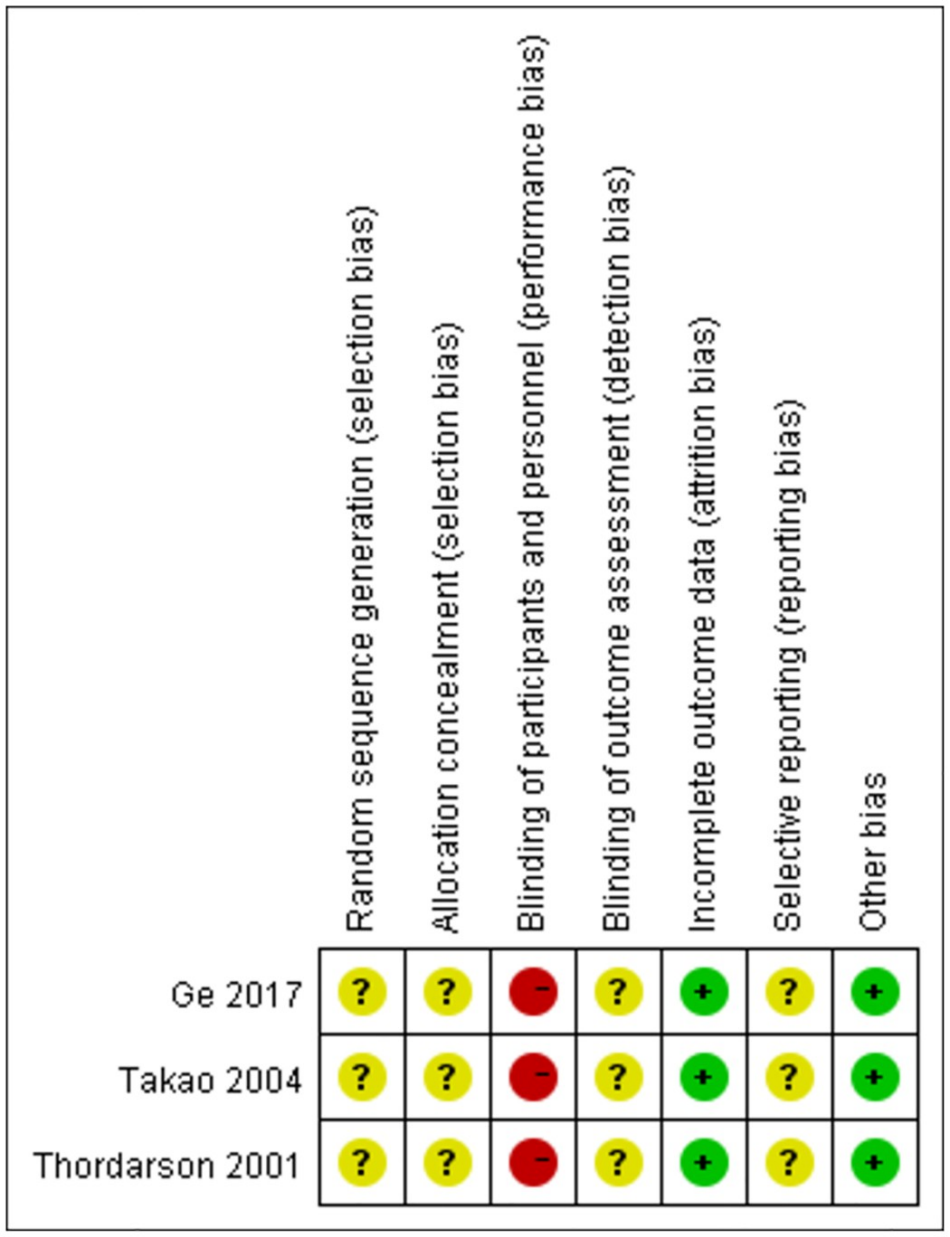
Methodological quality of RCTs.

Only three trials [23, 25, 26] were described as RCTs. The methodological quality results of the RCTs are depicted in Fig 2. No studies reported the methods for random sequence generation and allocation concealment. Due to the nature of operative treatments, it was difficult to be achieved by participant and personnel blinding. No trials reported the blindness for outcome assessments. It was difficult to assess the reporting bias because of the lack of protocols.

#### Non-randomized comparative studies

The methodological quality of the non-randomized comparative studies [14, 19–22, 24, 27] with the MINORS scores is presented in Table 2. The mean MINORS scores were 16.29 ± 2.05.

**Table 2:** Results of the MINORS evaluation.

Scale Item	Angthong 2016	Baumbach 2021	Chiang 2019	Fuchs 2016	Liu 2020	Smith 2020	Turhan 2013
1. A clearly stated aim	2	2	2	2	2	2	2
2. Inclusion of consecutive patients	2	2	2	2	2	2	2
3. Prospective collection of data	0	2	0	0	0	0	0
4. Endpoints appropriate to the aim of the study	1	2	2	2	2	2	2
5. Unbiased assessment of the study endpoint	0	0	0	0	0	0	0
6. Follow-up period appropriate to the aim of the study	0	2	2	2	2	2	2
7. Loss to follow up less than 5%	0	0	2	2	1	0	2
8. Prospective calculation of the study size	0	0	0	0	2	0	0
9. An adequate control group	2	2	2	2	2	2	2
10. Contemporary groups	2	0	0	2	2	2	2
11. Baseline equivalence of groups	1	2	2	1	2	2	2
12. Adequate statistical analyses	2	2	2	2	2	2	2
13. Total scores	12	16	16	17	19	16	18

### Functional outcomes

Functional outcomes were reported in nine included studies [14, 20–27]. Baumbach et al. [20] reported significantly better Foot and Ankle Ability Measure (FAAM) scores in the ARIF group than those in the ORIF group (p = .034). Ge et al. [23] observed significantly greater McGuire scores in the ARIF group than those in the ORIF group (p < .001). Smith et al. [24] noted significant improvements in PROMIS for Weber B fibula fractures in the ARIF group than in the ORIF group (p = .012). Fuchs et al. [22] reported no significant difference regarding PROMIS physical function and pain interference scores between patients in the ARIF group and ORIF group (p = .23 and p = .56, respectively). Thordarson et al. [26] noted no significant difference regarding SF-36 and Foot and Ankle MODEMS scores between patients in the ARIF group and ORIF group.

#### Olerud–Molander Ankle Score (OMAS)

**Fig 3:**
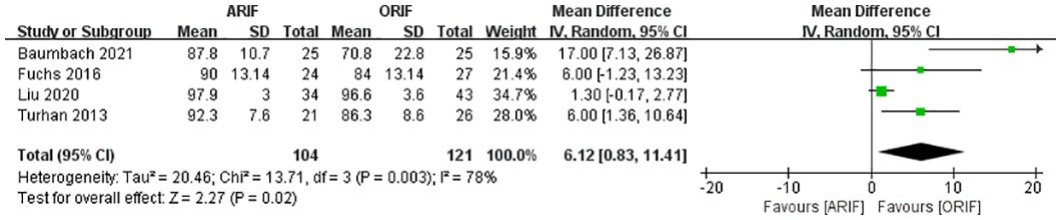
Forest plot of OMAS. There was a significant difference in OMAS between the two groups.

This score was reported in four included studies [14, 20, 22, 27]. A meta-analysis with the random-effects model (Fig 3) demonstrated that the OMAS value was statistically superior in the ARIF group (MD, 6.12 [95% CI, 0.83, 11.41]), with high heterogeneity (I^2^: 78%).

#### American Orthopaedic Foot and Ankle Society (AOFAS) Ankle—Hindfoot score

**Fig 4:**
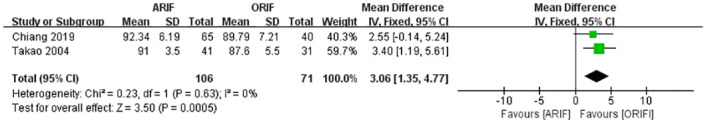
Forest plot of AOFAS ankle-hindfoot score. There was a significant difference in AOFAS between the two groups.

Two studies [21, 25] contributed data for analysis of the AOFAS ankle–hindfoot score. A meta-analysis with the fixed-effects model (Fig 4) revealed that the AOFAS ankle–hindfoot score was statistically superior in the ARIF group (MD, 3.06 [95% CI, 1.35, 4.77]), with no heterogeneity (I^2^: 0%).

### Post-operative complications

**Fig 5:**
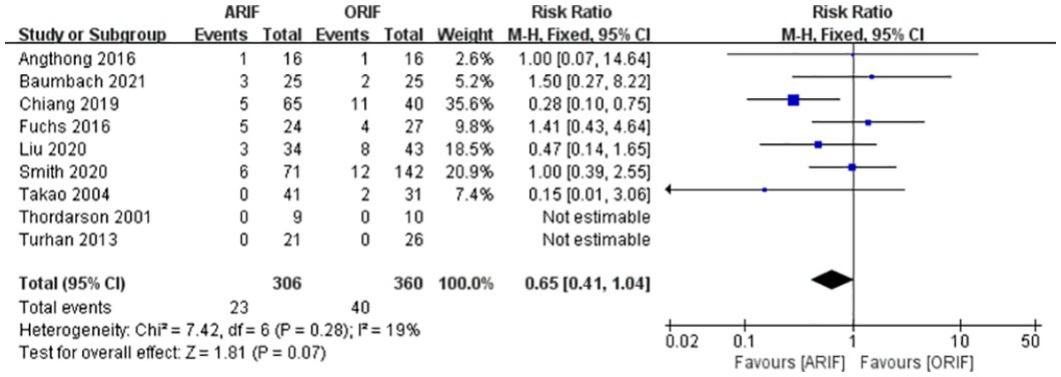
Forest plot of complications. There was a significant difference in complications between the two groups.

Nine included studies [14, 19–27] reported complications, including wound problems, fracture malalignment, lateral hardware irritation, infection, nerve injury, nonunion, and residual numbness. A meta-analysis with fixed-effects model (Fig 5) found no significant difference in the post-operative complication rate between the ARIF and ORIF groups (RR, 0.66 [95% CI, 0.41, 1.06]), with low heterogeneity (I^2^: 22%).

### Arthroscopic findings

Arthroscopic findings were reported in six included studies [14, 19, 22, 24, 25, 27] involving 225 patients, among whom a 62.2% (140/225) rate of evidence of chondral or OCL was reported. Three included studies [14, 19, 25] reported a 67.0% (61/91) rate of ankles with ligamentous injuries. A 26.1% (35/134) rate of loose bodies were found in three included studies [22, 24, 27].

### VAS score

**Fig 6:**
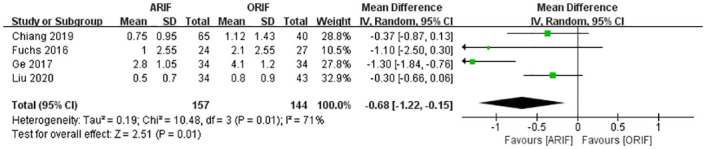
Forest plot of VAS. There was a significant difference in VAS between the two groups.

Four included studies [14, 21–23] contributed data for analysis of the VAS score. A meta-analysis with the random-effects model (Fig 6) showed that the VAS score was statistically superior in the ARIF group (MD, −0.68 [95% CI, −1.22, −0.15]), with high heterogeneity (I^2^: 71%).

### Operation time

**Fig 7:**
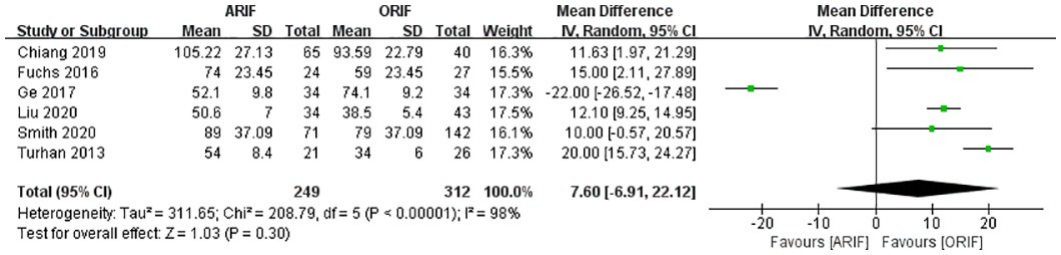
Forest plot of the operation time. No significant difference in the operation time between the two groups.

The operation time was reported in six included studies [14, 21–24, 27]. A meta-analysis with the random-effects model (Fig 7) detected no significant difference in the operation time between the ARIF and ORIF groups (MD, 7.60 [95% CI, −6.91, 22.12]), with high heterogeneity (I^2^: 98%).

### Publication bias

**Fig 8:**
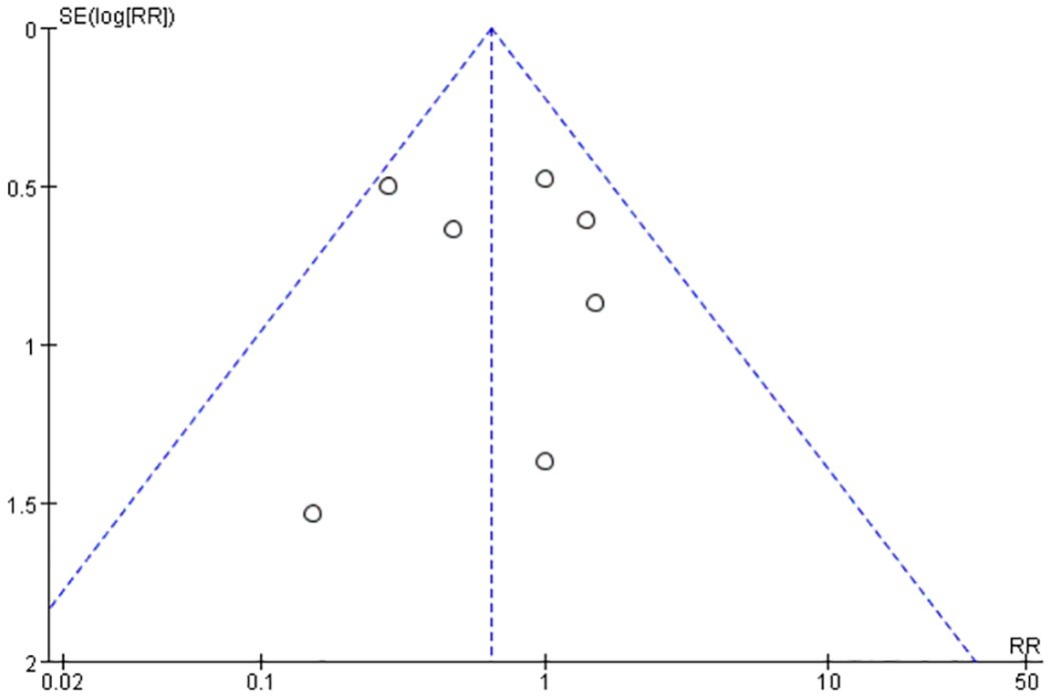
Funnel plot.

Funnel plot analysis of seven included studies reporting the complication rate is presented in Fig 8. The distribution of the left and right sides in these studies was basically symmetrical, indicating no significant publication bias.

## Discussion

Our systematic review and meta-analysis revealed that ARIF for ankle fractures was superior regarding functional outcomes and VAS scores when compared with ORIF. No significant difference was detected in the post-operative complication rate and operation time between the ARIF and ORIF groups. A high incidence of OCL, ligamentous injuries, and loose bodies with ankle fractures was found by ankle arthroscopy.

Several studies have reported that occult intra-articular injury may be the cause of unsatisfactory outcomes in ankle fractures [25, 28–31]. As an adjunct to the treatment of ankle fractures, ankle arthroscopy can be used as a diagnostic and prognostic tool, which is helpful for addressing intra-articular disorders and plays a role in preventing posttraumatic osteoarthritis [32, 33]. Baumbach et al. [20] reported that the OMAS and FAAM Activities of Daily Living were statistically significant in the ARIF group compared with the ORIF group over a four-year follow-up period. They also noted that more patients in the ARIF cohort returned to sport and had a higher FAAM Sports score. A retrospective cohort study by Smith et al. [24] performed a subgroup analysis to compare the outcomes among Weber B ankle fractures in the ARIF and ORIF groups during a mean follow-up of 32.4 months. They reported that the PROMIS physical function score and satisfaction rate were statistically higher in those patients who underwent arthroscopy compared with ORIF alone. Ceccarini et al. [2] reported the effectiveness of ankle arthroscopic debridement in acute or subacute ankle fractures. They found that ankle arthroscopy could improve the clinical outcome and offer an essential option for patients both in acute and in sequelae after an ankle fracture. Kim et al. [1] reviewed second-look arthroscopic findings and evaluated clinical outcomes in 40 ankles. In total, 17 cases were reported as newly discovered chondral lesions in secondary arthroscopy. The authors believed that both initial and secondary arthroscopy combined with treatment of intra-articular lesions may improve clinical outcomes. In our meta-analysis, we found a significant difference in the OMAS and AOFAS ankle–hindfoot scores between the ARIF group and ORIF group. Meanwhile, the VAS score was statistically superior in the ARIF group compared with the ORIF group. We believe that ARIF can improve both functional outcome and pain score by treatment of intra-articular lesions, synovitis, ligamentous injuries, and loose bodies.

The concerns about longer operative time and possible additional complication rate had impeded generalized use of ARIF for ankle fractures [5]. Although no operative complications associated with ARIF have been reported, the complications during ankle arthroscopic procedures might occur such as superficial peroneal neuritis, damage to the neurovascular bundle, and wound problems [34]. In our meta-analysis, no significant difference was found in the post-operative complication rate between the ARIF and ORIF groups. Some authors even found that ARIF may effectively decrease the incidence of complications and reoperations in ORIF [21]. The operation time of ARIF is 11–20 min longer than that of ORIF in previous studies [21, 27]. However, we found no significant difference in the operation time between the ARIF and ORIF groups. Smith et al. [24] also found that the difference of operation time was not statistically significant between the two groups. Ge et al. [23] noted the operation time of the ARIF group was significantly reduced. The learning curve of ARIF and the different fracture types may be the reason for the difference in the operation time. Experienced surgeons and simpler fractures are likely to shorten the surgical time. Meanwhile, arthroscopically assisted reduction with a fibular intramedullary nail or a percutaneous plate will further reduce the time spent on suturing the wound [35]. With the increase in surgical experience, the occurrence of post-operative complications and the extension of operation time will no longer be a potential concern for surgeons.

The advantage of ARIF can help us identify the occult intra-articular disorders during the initial fractures. Williamson et al. [13] reported that almost 55% of patients with acute ankle fracture had concomitant OCLs affecting the ankle joint. In our study, we found that a 62.2% (140/225) rate of evidence of OCL at the time of arthroscopy for ankle fractures. Although the incidence is variable in different studies, ankle arthroscopy as a diagnostic tool has shown a high sensitivity for detection of OCLs [11, 36, 37].

The concomitant stabilization of the syndesmosis is necessary because its misdiagnosis or inadequate treatment may lead to persistent pain, dysfunction, and early osteoarthritis [38]. Some authors believe the repair of the deltoid ligament patients with acute ankle fractures might be beneficial to ankle joint stability and assist in improving the quality of ankle reduction [39]. The assessment of syndesmosis integrity and medial clear space on plain radiograph was shown to be inaccurate [40]. In our study, we found that 67.0% (61/91) of ankles had ligamentous injuries. A three-dimensional assessment of the syndesmosis using ankle arthroscopy can help assure the anatomical reduction of the syndesmosis. Arthroscopy can avoid the false impression of widened medial joint space on fluoroscopy due to congenital anomaly [40]. We believe that ARIF can guide the anatomical reduction and take ligamentous injuries into consideration.

A meta-analysis by Lee et al. [5] reported that ARIF was more beneficial with respect to functional outcomes when compared with ORIF. This study only included four studies and summarized effect size based on different outcome measure tools. Williams et al. [41] performed a systematic review on ARIF of foot and ankle fractures. The authors argued that despite limited evidence, arthroscopy is expected to become a valuable tool for internal fixation of foot and ankle fractures. A systematic review by Williamson et al. [13] reported the incidence of OCLs in ankle fractures identified with ankle arthroscopy. They found a high incidence (54.5%) of intra-articular chondral lesion in the setting of rotationally unstable ankle fractures as demonstrated by arthroscopy. In our updated meta-analysis, we included 10 studies and summarized effect size based on the OMAS and AOFAS. We found that the ARIF group showed statistically significant differences in functional outcomes and VAS score during the mid-term follow-up period. The high incidence of osteochondral lesion and ligamentous injurie in acute ankle fractures was demonstrated by ankle arthroscopy.

This study still has several limitations. First, the number of included RCTs was small. It is debatable whether routine arthroscopy is cost-effective at the time of ORIF [25, 42, 43], so RCTs were difficult to perform. Second, due to the exclusion of articles not written in English, some articles may have been omitted. Third, the observed heterogeneity might have contributed to differences in fracture type, age, and experience of the surgeons.

## Conclusion

ARIF for ankle fractures might be beneficial to offer superior functional outcomes and VAS score compared with ORIF. Orthopedic surgeons should take a high incidence of OCLs and ligamentous injuries into consideration for treatment of acute ankle fractures. We believe that with the increase in surgical experience, the occurrence of post-operative complications and the extension of operation time will no longer be a potential concern for surgeons.

## Supporting information

S1 Checklist PRISMA 2009 checklist. (DOCX)
